# Effect of a Simulated Coal Mine Environment on Polyurethane Grouting Material and a Proposed Polyurethane Strengthening Method

**DOI:** 10.3390/polym15224449

**Published:** 2023-11-17

**Authors:** Kai Hou, Shuai Wang, Xin Yao, Shun Yao, Xinxing Zhou, Jianchao Ma, Pengfei Wang, Guorui Feng

**Affiliations:** 1 College of Mining Engineering, Taiyuan University of Technology, Taiyuan 030024, China; kaihou2015@163.com (K.H.); wangshuai1277@link.tyut.edu.cn (S.W.); yaoxin1291@link.tyut.edu.cn (X.Y.); y0aoshun1290@link.tyut.edu.cn (S.Y.); wangpengfei@tyut.edu.cn (P.W.); 2 Key Laboratory of Shanxi Province for Mine Rock Strata Control and Disaster Prevention, Taiyuan University of Technology, Taiyuan 030024, China; 3 Key Laboratory of Highway Construction and Maintenance Technology in Loess Region of Ministry of Transport, Shanxi Transportation Technology Research & Development Co., Ltd., Taiyuan 030032, China

**Keywords:** polyurethane, gangue particle size, mineral grouting, ultrasound

## Abstract

When it comes to grouting in coal mines, polyurethane (PU) is often utilized. However, it is of vital importance to consistently improve the mineral PU, considering the significant amount of environmental deterioration to which it is prone. Laboratory experiments were used to model various coal mine conditions. Additionally, a workable technique for PU strengthening using ultrasonic waves was proposed. Compression tests and scanning electron microscopy (SEM) were used to describe the PU–gangue material’s induration characteristics. The results showed that ultrasound has a positive impact on PU’s mechanical strength. The final strength of the PU was significantly impacted by the size of the coal gangue particles, the amount of dust, and the amount of water. The induration made of gangue and PU with the same mass but differing particle sizes was noticeably different in its compressive strength. The strengthening mechanism showed that the average size of the rigid foam after the ultrasound treatment was smaller, and the ‘honeycomb’-structured space in the inner section was more compact, resulting in the rigid PU foam having a higher compressive strength after ultrasound treatment. Furthermore, the dust content and water content of coal mines need to be controlled within a specific range to ensure the effective use of PU grouting materials.

## 1. Introduction

Grouting technology, which plays a significant role in the field of green mine construction, has been widely utilized in mine engineering as an effective means to seal water, stabilize ground, heat insulation, and repair cracks [1]. Over the past few decades, distinct grouting materials have been developed, such as waterglass [2], silica sol [3], cement [4], PU, epoxy resins [5], and their compounds [6]. Given its excellent synthetic properties, such as its outstanding chemical stability [7], large expansion rate [8], high mechanical strength [9], corrosion resistance [10], self-extinguishing flame retardancy [11], low environmental pollution risk, low permeability [12], and fast reaction times, PU grouting has been widely applied as a sealing and plugging method to prevent mine disasters [13]. An inorganic filler is usually added in order to improve the performance of PU grouting materials and reduce their cost.

Coal gangue is a type of solid mining waste, more than 1 billion tons of which are in storage in China. Its main components are silicon dioxide (SiO_2_), aluminum oxide (Al_2_O_3_), iron oxide (Fe_2_O_3_), calcium oxide (CaO), magnesium oxide (MgO), and a small number of other oxides. At present, coal gangue has become one of the main potential filler materials in polymer matrices due to a number of advantages. Jin et al. [14] observed that it has good interaction performance, thanks to its physical properties. Liu et al. [15] reported that coal gangue, after microstructure adjustment, can promote stable structures in asphalt for durable pavements. Additionally, Zhang et al. [16] proposed a new type of coal gangue geopolymer and found that the addition of coal gangue significantly improved the mechanical strength of the geopolymer. The authors of [17] described the dispersibility of kaolinite-rich coal gangue in a rubber matrix and the mechanism by which coal gangue enhanced the mechanical properties and thermal stability of styrene butadiene rubber. All of these studies on coal gangue composites confirm that it can improve the mechanical properties of polymer materials. However, it is rare to find studies on the effects of coal gangue on the mechanical properties and surface relationships of PU grouting materials.

The mechanical effect [18], cavitation effect [19], thermal effect [20], and chemical effect [21] of ultrasonic waves can bring about significant improvements in the physical and chemical properties of materials; therefore, ultrasonic waves are widely used in the synthesis processing of polymers. However, the utilization of ultrasound with PU grouting materials is still a barren research field. On the other hand, the dispersion of nanoparticles in PU foam containing silicone aerogel and glass bubbles was enhanced by ultrasonic dispersion [22]. For example, Park et al. [23] designed a microporous PU film and used ultrasound during the coagulation stage. The film that was made using both ultrasound and normal processes was prominently different in terms of its mechanical and transport properties. However, there has been little research on the ultrasonic treatment of PU grouting materials. 

Because a coal seam is affected by the mine’s pressure and geological structure, a certain amount of primary mine dust is produced and contained in the fissures of the coal seam and the surrounding rock [24]. In the various links of the coal production chain, such as mining and transportation, secondary mine dust and water are generated by the cutting, crushing, and loading and unloading of coal and rock masses [25]. In the process of grouting, the grouting materials are inevitably exposed to pores containing dust and water. It is necessary to accurately understand the influence of mine dust and water on PU grouting materials.

In this study, the different conditions that exist in coal mines were simulated in the laboratory. The performance in terms of the particle size, dust content, and water content of broken gangue–PU was assessed. Ultrasound strengthening of PU at the foaming stages was carried out, and compressive strength measurements and SEM were performed for composite characterization. The adaptability of the PU grouting material to the gangue material under different circumstances was discussed.

## 2. Materials and Methods

### 2.1. Materials

The polyether polyol, polyester polyol, silicone oil, catalyst, and fire-retardant mass were provided by Shanghai DONGDA Polyurethane Co., Ltd. (Shanghai, China). The 4,4′-diphenylmethane-diisocyanate (MDI) with 99% purity and a density of 1.19 (25 °C, g/cm^3^) was purchased from Shanghai BASF Polyurethane Co., Ltd. (Shanghai, China). The deionized water was provided by our laboratory. The coal gangue was collected from the Tunlan Coal Mine (Taiyuan, China). Furthermore, the coal gangue was obtained by screening in order to obtain different particle sizes (0–5, 5–10, 10–15, 15–20 mm), and was cleaned afterwards to remove coal dust. The gangue was obtained via drying for 24 h at a low temperature of 40 °C. In this operation, all reagents were directly used without further purification.

The PU grout selected for this study was a bicomponent, expansive grouting material. Component A consisted of polyether polyol, polyester polyol, silicone oil, a catalyst, and a fire-retardant mass at a ratio of 15:40:1:4:5. Component B consisted of 4,4′-diphenylmethane-diisocyanate (MDI). The physical properties of the gangue are shown in Table 1.

### 2.2. Fabrication of Conventional PU and Ultrasound-Treated PU

**Figure 1:**
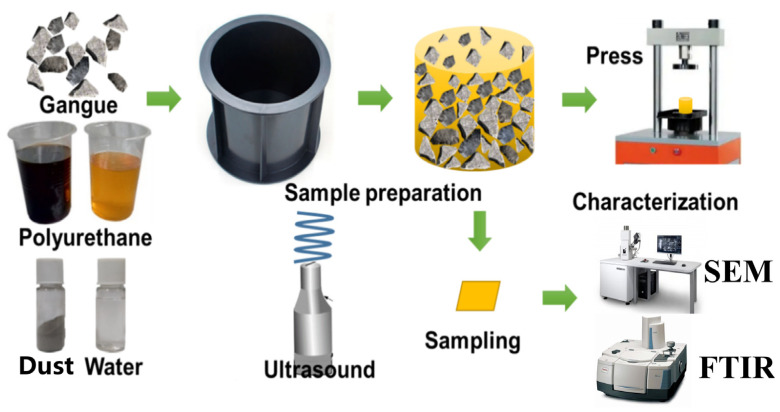
Sample preparation, experimentation, and characterization of the PU grouting materials.

The PU grout selected for this study was a bicomponent, expansive grouting material. Component A1 consisted of polyether polyol, polyester polyol, silicone oil, a catalyst, and a fire-retardant mass at a ratio of 15:40:1:4:5 and with a density of 50 kg/m^3^. Component A2 consisted of polyether polyol, polyester polyol, silicone oil, a catalyst, and a fire-retardant mass at a ratio of 10:50:1:4:5 and with a density of 100 kg/m^3^. Component B consisted of 4,4′-diphenylmethane-diisocyanate (MDI). The PU composites were manufactured according to the schematic diagram shown in Figure 1. The PU components A1, A2, and B were added to a beaker at a mass ratio of 1:1 and poured into a cylindrical plastic mold with a cover (height of 50 ± 1 mm and diameter of 50 ± 0.1 mm) after rapid stirring with a glass rod. The die was kept in a closed state throughout the experiment. Each group of materials was foamed three times. After the PU foaming was finished, the numbered materials were placed at a normal temperature (25 °C) and atmospheric pressure for 168 H, which was followed by a pressure test. The samples of components A1 and B were designated O-50 and S-50 (after ultrasound). The samples of components A2 and B were designated O-100 and S-100.

### 2.3. Fabrication of PU–Gangue Material

The coal gangue (100.0 g) was placed in the mold in advance. The O-100 material was prepared in a cylindrical mold with a lid. According to the particle size of the gangue added, the different labels were G-A (no gangue), G-B (0–5 mm), G-C (5–10 mm), G-D (10–15 mm), and G-E (15–20 mm).

### 2.4. Fabrication of the Dustiness of PU

The dust was pre-mixed with component A1. The O-100 material was prepared in a cylindrical mold with a lid. According to the amount of dust content added, the different labels were d-0 (no dustiness), d-5% (5 wt%), d-10% (10 wt%), d-15% (15 wt%), d-20% (20 wt%), and d-25% (25 wt%).

### 2.5. Fabrication of Water–PU

Firstly, components A2 and B were added in a 1:1 ratio to the beaker. Secondly, different amounts of water were added by quickly stirring and pouring the water into the mold foaming. Depending on the amount of water added, the different labels were W-0 (no water), W-1% (1 wt%), W-2% (2 wt%), W-4% (4 wt%), and W-5% (5 wt%).

### 2.6. Mechanical Tests and Characterization

The mechanical test was performed on a universal testing machine (WDW-100, Changchun New Testing Machine Co., Ltd., Changchun, China). The compressive strength was measured to determine the effects of the different conditions of the grouting materials.

In the compression test following the procedure of GB/T 1041–2008 [26], foam with dimensions of Φ50 (±0.1) mm × 50 (±0.1) mm was compressed to 50% of its original thickness, and the compression speed was 5 mm/min. Each group of samples was tested five times to determine the average value, and the error was also estimated. The stress–strain curves of the grouting materials were obtained. The modulus of elasticity was calculated from the obtained stress–strain curve.

Ultrasound (KQ–250DE, Shanghai Shumei Ultrasonic Instrument Co., Ltd., Shanghai, China) with an input power of 250 W was used. SEM (ZEISS Gemini 300, Carl Zeiss AG, Jena, Germany) was used to observe the topography of the PU composites with an acceleration voltage of 10 kV and a magnification of 100. The test temperature was 23 ± 2 °C, and the samples were sliced. 

Fourier transform infrared spectroscopy (FT–IR) was carried out using the Bruker (INVENIO S, Bruker Corporation, Coventry, UK), where the scanning wavenumber was 4000~400 cm^−1^, and the PU samples were prepared using a KBr disc.

## 3. Results

### 3.1. Effect of Ultrasound on the PU Foam

Firstly, a foaming experiment was carried out in the state of natural foaming. The A and B PU materials reacted within one minute after mixing and the volume change remained stable after five minutes. The volumes of S-50 and O-50 were measured after they became stable. The volume of S-50 was 7.50% smaller than that of O-50. The volume of S-100 was 4.30% smaller than that of O-100.

**Figure 2:**
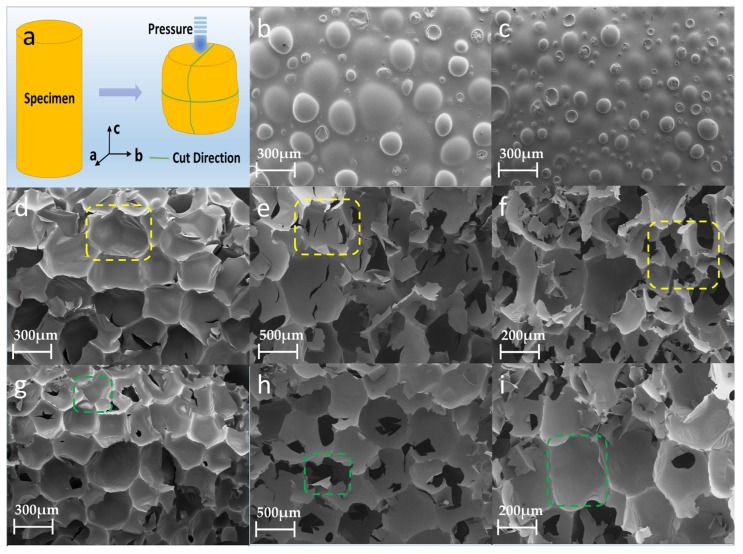
SEM images: (**a**) compression diagram of PU; (**b**) PU outer surface; (**c**) ultrasonic PU outer surface; (**d**) PU cut noodles; (**e**) compressed PU longitudinal section; (**f**) compressed PU cross-section; (**g**) ultrasonic PU cut noodles; (**h**) compressed ultrasonic PU longitudinal section; (**i**) compressed ultrasonic PU cross-section.

Figure 2 shows the morphological and structural information for the PU samples prepared using the conventional method and ultrasonic method. Figure 2a shows the compression process and section direction of the prepared PU grouting material. As shown in Figure 2b,d, the pore structure of the pure PU is complete, the pore wall surface is smooth, the pore size is relatively uniform, and the pore sizes are mainly distributed within the range of 100–500 μm. As shown in Figure 2c,g, the ultrasonic PU has a uniform pore size, complete structure, and smooth pore walls, while the pore sizes are mainly distributed with the range of 50–300 μm, which means that the pore size distribution is smaller and the pore sizes are more uniform than that of the PU prepared with conventional methods. This is because the ultrasonic waves improve the compatibility between the PU components, which makes the bonding interface of each component stronger and increases the physical crosslinking degree of the PU [27]. In addition, due to the violent vibration in the liquid particles, when ultrasonic waves propagate in the liquid, small holes with liquid are generated inside these particles. These cavities rapidly expand and close, causing violent collisions between the liquid particles that create a pressure measuring in the thousands to tens of thousands, and the volume of the bubble is reduced. This violent interaction between particles will make the temperature of the liquid rise suddenly, which plays a role in the stirring process and increases the density of the PU, leading the PU to obtain better mechanical properties [28]. This was also reported by Yu et al. [29]. SEM images of the compressed PU are shown in Figure 2e,f,h,i. In the longitudinal direction, after the irregular bubble is compressed into a sphere, the microscopic performance is determined based on the bending deformation of the bubble wall and the mutual extrusion between the adjacent bubble holes. Among them, the PU prepared with the conventional method has more cracks (inside the yellow dotted line). In the horizontal direction, it was microscopically observed that the buckling and bending of the bubble wall causes the bubble to break and collapse (inside the green dotted line).

**Figure 3:**
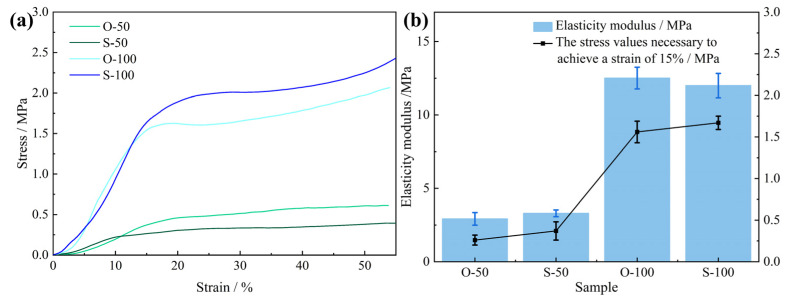
Stress–strain curves (**a**) and elasticity moduli (**b**) of rigid PU foam samples under different treatment conditions and densities.

Figure 3 shows the compressive properties of the PU foam materials prepared with diverse treatment conditions and densities. The foam was cut into Φ50 mm × 50 mm sample strips to facilitate the testing of the compressive properties. The compressive stress–strain curves of the test results are shown in Figure 3a. After compression, the stress–strain curves of the rigid PU foam materials under different treatment conditions and densities included the linear elastic stage, plastic platform stage, and densification stage. In the linear elastic stage, with the increase in strain, the stress increases linearly, and the elastic behavior in this stage is mainly dictated by the cellular structural strength. The elastic moduli of O-100 and S-100 are obviously greater than those of O-50 and S-50. In the yield plateau stage, the stress remains unchanged or increases slowly with the increase in strain, and a long yield plateau appears, which is plastic deformation. The microscopic manifestation demonstrates the bubble breakage and collapse caused by the buckling and bending of the bubble wall. The plastic plateau stage appears earlier in the conventional conditions for O-50 and O-100 compared to S-50 and S-100 at the same density. The strength and plateau stress of the PU rigid foam material treated by ultrasound are increased by 102.98% and 71.58%, respectively, at a density of 50 kg/m^3^. When the density is 100 kg/m^3^, the strength of the rigid PU foam material treated by ultrasound is increased by 23.00% and the plateau stress is increased by 24.84%. Figure 3b shows the elasticity modulus and stress value of the control PU and the PU after ultrasound. The effect of ultrasonic waves on the 50 kg/m^3^-density PU foam is more obvious, whereby the elastic modulus and compressive strength are increased, whereas their effect on the 100 kg/m^3^-density PU foam is less obvious.

The above analysis shows that the average size of the rigid foam after the ultrasound treatment is smaller and the ‘honeycomb’-structured space of the inner section is more compact, resulting in a higher compressive strength for the rigid PU foam after ultrasound.

### 3.2. Effect of Gangue Granularity on PU–Gangue Induration

**Figure 4:**
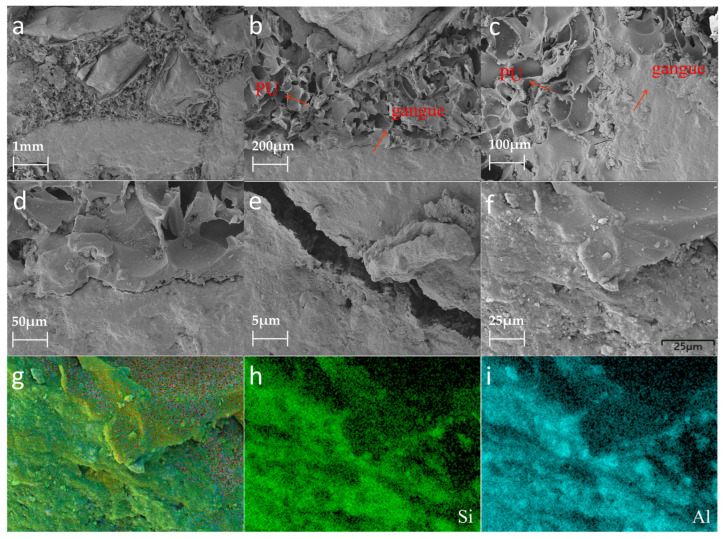
SEM images: (**a**–**e**) PU–gangue 5–10 mm; (**f**–**i**) corresponding elemental mapping of the PU–gangue material.

Figure 4 shows the morphological and structural information for the PU–gangue material. Figure 4a–c shows that the PU–gangue material was observed at different magnifications, in which the gangue particle size range was 5–10 mm. The gangue was found to be unevenly dispersed in the PU matrix. During the foaming process, the organic–inorganic interface visible in the PU matrix easily separates and forms cracks. This finding suggests that PU has poor interfacial compatibility with gangue. However, the interface relationship between PU and gangue can be clearly observed in the EDS images presented in Figure 4f–i, which could represent PU-coated gangue or Si–O-Si-formed PU and gangue.

As shown in Figure 4, the experiments were carried out using mold confinement. In this case, the PU was naturally foamed and cured in the initial stage. However, in the later stage of foaming, as the mold was in a semi-closed state, the PU was confined by the mold; the foam was squeezed and the pore size was redistributed. This state is similar to the actual grouting environment of a coal mine. After adding coal gangue with the same mass and different particle sizes, the induration strength increased significantly because the PU and rocks of different particle sizes formed a ‘composite material’. Together, they ‘resist’ the external pressure, in which PU acts as a cementitious material and coal gangue acts as an aggregate.

**Figure 5:**
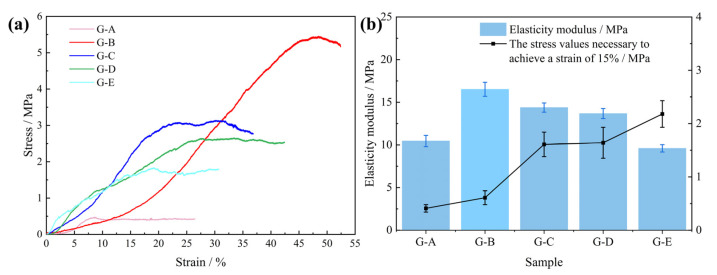
Stress–strain curves (**a**) and elasticity moduli (**b**) of rigid PU foam samples with different gangue particle sizes.

Although the coal gangue mass stays the same, the apparent volume is dependent on the coal gangue’s particle size. The gangue particle size and porosity greatly influence the PU grouting material [30]. Coal gangue with a larger particle size exhibits a more obvious skeleton effect, which shows that the compressive strength of the composite increases significantly in the linear elastic segment. When the size of the coal gangue is in the range of 5–20 mm, the compressive strength of the PU–gangue induration decreases when the particle size increases. This is attributed to the improved framework stability and coal gangue particle size decreasing [31]. The initial compressive strength of the PU–gangue induration with particle sizes in the range of 0–5 mm increases slowly, although the later strength is high. The initial strength of the induration with a particle size range of 0–5 mm is lower and the curve is smoother because the apparent volume of coal gangue with a smaller particle size is smaller at the same mass. Thus, the PU occupies a larger residual volume after foaming in a closed space. The initial compressive strength is mainly supported by PU, and it is weak over a strain range of 15–20%. The curve continues to rise, which means that the skeleton effect begins after the contact with and compaction of the coal gangue [32]. Within a specific range, the skeleton stability of coal gangue with a smaller particle size improves and the curve of the later growth part is smoother [33]. Figure 5b shows the elasticity modulus and stress value of the PU–gangue composite material. An appropriate addition of gangue can significantly improve the elastic modulus and compressive strength, and the increase in elastic modulus is related to the particle size of the gangue, among which the 0–5 mm gangue has the best effect. This is consistent with the results from the stress–strain curve.

### 3.3. Effect of Dustiness on PU Induration

**Figure 6:**
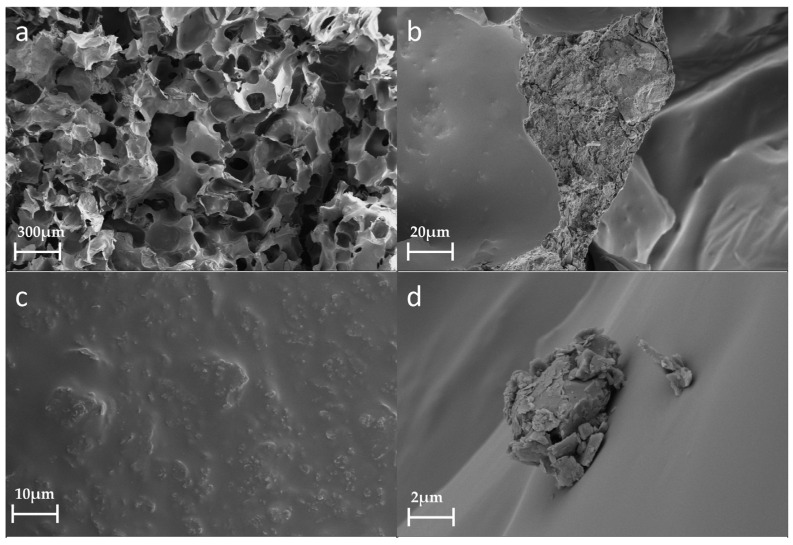
SEM images of PU foam containing 20% dust: (**a**) 30×; (**b**) 500×; (**c**) 1500×; (**d**) 5000×.

Figure 6 shows the morphological and structural information for PU foam containing 20% dust. Compared with the SEM results for pure PU, the section morphology of the induration containing 20% dust is more random (Figure 6a). The addition of excessive coal dust disturbs the inherent cellular structure of the PU foam [34], despite the cellular size of this structure being smaller than that of pure PU. The coal dust particles enter the cross-section of the PU foam (Figure 6b,c). The coal dust is dispersed evenly in the PU matrix, which smoothens their structures, making the surface rougher despite having a relatively smooth fracture surface. Some of the coal dust does not enter the PU wall but adheres to it, and the coal dust itself appears as irregular aggregates (Figure 6d). There may be intermolecular forces (van der Waals forces) among the coal dust particles and between the coal dust and PU.

**Figure 7:**
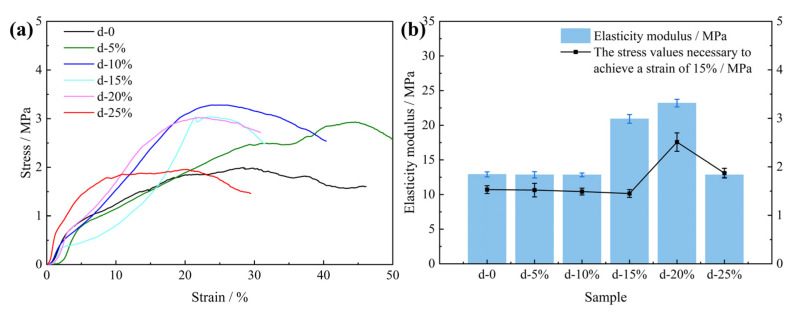
Stress–strain curves (**a**) and elasticity moduli (**b**) of rigid PU foam samples with different dust contents.

Figure 7 shows the compressive properties of the PU composite foam. The ultimate compressive strength of the PU–gangue induration increased with the increasing content of the dust particles, given that the dust content was <10% (Figure 7). Meanwhile, the peak stress of the induration decreased when the amount of filler exceeded this critical value. The above phenomena occur in many PU matrices that contain inorganic micro-nano filler particles. The pore size of the PU foam honeycomb structure decreases with increasing filler content. Hence, its structure becomes more compact, which causes an increase in the ultimate compressive strength (peak stress) macroscopically [35]. On the other hand, if the filler is excessive, the cellular structure of the PU foam will be distorted and deformed with less homogeneity, which is caused by a decrease in the ultimate compressive strength in the macrocosm. However, these artificially added inorganic micro-nano fillers are different from natural coal dust because they contain specific functional groups. Studies have shown that the thermal stability of PU containing nano-composites is improved significantly by the addition of these micro-nano fillers [35]. Additionally, certain fillers may provide the PU with other functions. For example, the thermal conductivity of PU was improved by 332% with a 10 wt% addition of functionalized multi-walled carbon nanotubes [36]. Synthesized PU-AT/TiO_2_-ZrO_2_ nanoparticles also enable PU to show better performance and prolong its service life [37]. Figure 7b shows the elasticity modulus and stress values of the PU composite materials with different dust contents. When the dust content range is 15–20%, the elastic modulus and compressive strength of the composite can be significantly improved. Excessive dust has little effect on the elastic modulus, which can be attributed to the broken PU cell structure. This is consistent with the stress test results.

### 3.4. Effect of Water on PU Induration

**Figure 8:**
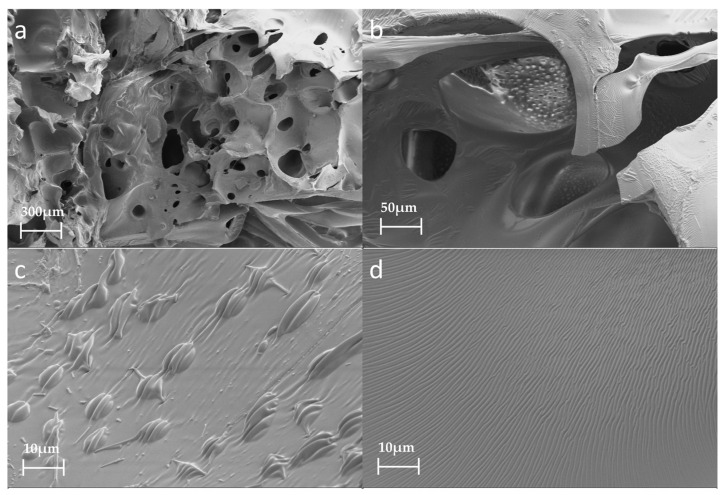
SEM images of PU foam containing 5% water: (**a**) 30×; (**b**) 200×; (**c**) 1000×; (**d**) 1000×.

The average cell size in the foams increased with increasing water content in the range of 0–3%, resulting in reduced foam strength [38]. However, when the water content increased to 5%, small bubble holes appeared on the inner walls of the larger bubble holes and the cell size was not uniform (Figure 8a,b). Protruding cell bodies (Figure 8c) and lines (Figure 8d) also appeared on the smooth cell walls. The possible reasons for this are as follows. With the increasing water content, the active generation of CO_2_ gas increases due to the large amount of water reacting with isocyanate [39]. Small cell cavities and cell bodies that protrude from the PU surface are generated inside the large cell cavity. In addition, PU foaming is carried out in a closed container to simulate the movement and foaming process of PU in the cracks of the crushed coal gangue. When PU is foamed and reaches a particular stage, the structure of the PU will be squeezed, which will deform the cell cavity and form wrinkles on the surface of the cell wall. This also causes a significant decrease in the strength of the PU foam. 

**Figure 9:**
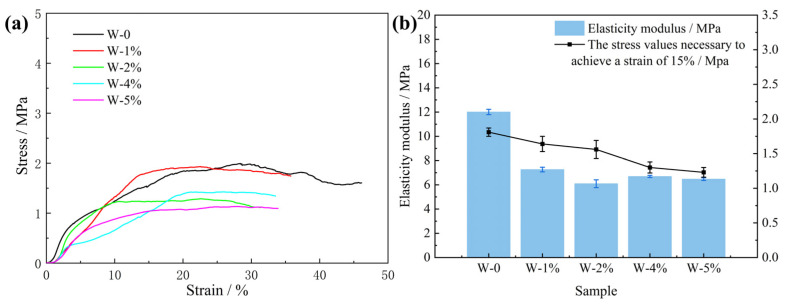
Stress–strain curves (**a**) and elasticity moduli (**b**) of rigid PU foam samples with different water contents.

Figure 8 shows the compressive properties of the characterized PU composite foam. The addition of water obviously affected the strength of the PU. With the increase in water, the PU strength decreased significantly. Figure 9 shows the elasticity moduli and stress values of the PU composite materials with different water contents. The addition of water significantly reduced the elastic modulus and compressive strength of the PU. Water can impart unique properties onto the PU. Figure 8d shows each slit, all of which have rough surfaces and a ditch-like structure, formed by the evaporation of water molecules [40]. In the PU foaming reaction, water is added as a special chemical foaming agent and reacts with isocyanate to form unstable carbamic acid, which is finally decomposed into amines and carbon dioxide (CO_2_), according to Reaction (1). The resulting amine reacts with the isocyanate to form a substituted urea (see Reaction (2)). Whether it is water in the air or directly added water, the two hydrogen atoms of the water molecule can react with the isocyanate group to produce carbon dioxide. The carbon dioxide gas diffuses into the already existing bubble and the foam rises as it increases in volume. At the same time, the viscosity of the mixture increases with polymerization and gelation [41]. Therefore, based on the chemical reaction, the addition of water undoubtedly affects the strength of the PU.

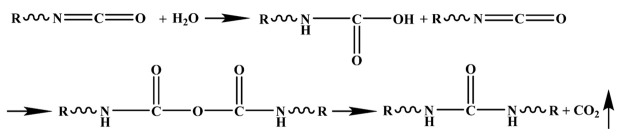
(1)
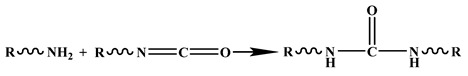
(2)

### 3.5. Effect of Water on the Characteristic Functional Groups of PU–Gangue Material

**Figure 10:**
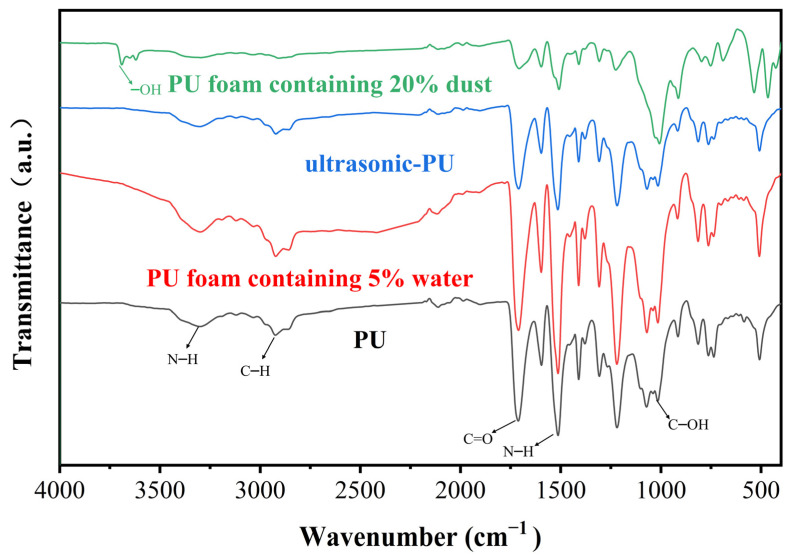
FTIR spectra of PU, PU foam containing 5% water, ultrasonic PU, and PU foam containing 20% dust.

As shown in Figure 10, the structure of PU was further analyzed using the FTIR spectrum. Table 2 lists the observed frequency bands representing the peak vibrations. For pristine polyurethane, the intense bands at 1306 and 1512 cm^−1^ are attributed to the C–N stretching vibration and N–H deformation, respectively. For ultrasonic PU, the peak intensity of the PU after ultrasonic treatment was significantly reduced, which was caused by the enhanced effect of the ultrasonic treatment on the material transfer and the more complete chemical reaction [42]. The ultrasonic waves improve the compatibility between the PU components, which makes the bonding interface of each component stronger and increases the physical crosslinking degree of PU [43]. For PU foam containing 20% dust, the absorption peak of C–OH was obviously enhanced at 1071 cm^−1^, while a new stretching vibration absorption peak of free hydroxy(–OH) appeared at 3691 cm^−1^. This can be attributed to the addition of coal dust, which introduces new functional groups (–OH) and makes the PU structure more compact. For PU foam containing 5% water, each absorption peak was slightly enhanced and no new absorption peaks were generated, indicating that water escapes as steam during the PU reaction, forming a larger bubble structure and reducing the mechanical properties of PU.

## 4. Conclusions

This study simulated the application of PU foam in a coal mine environment. The results led to the following conclusions:

(1) In the natural foaming stage of PU, the final volume of the PU can be reduced by introducing ultrasound waves. After the ultrasound treatment, the volume of the PU foam with a final density of 50 kg/m^3^ was reduced by 7.50%. However, the strength is increased by 102.98% and the plateau stress was increased by 71.58%. The PU foam material with a density of 100 kg/m^3^ was subjected to ultrasonic treatment. The strength and the plateau stress of the rigid PU foam material treated with ultrasound were increased by 23.00% and 24.84%, respectively. Ultrasound reduces the cavity volume of PU in the foaming stage and enhances the mechanical strength of PU. The introduction of ultrasonic waves in the foaming process makes it possible to further increase the strength of PU;
(2) The compressive strength of an induration composed of PU and coal gangue with the same mass but different particle sizes was significantly different than that of pure PU as it was considerably higher, which was due to the differences in the apparent density and structure of the coal gangue samples with different particle sizes;
(3) The induration performance is affected by dust in the mine’s grouting cracks. The dust will improve the strength of PU grouting materials over a certain content range. However, the content cannot exceed 10% of the mass of the PU or it will deteriorate the PU’s performance. If the dust content is above 20%, it will increase the disorder of the cross-section morphology of PU materials, both reducing the consistency and decreasing the strength of the PU;
(4) The PU’s performance is affected by water in the mine’s grouting cracks. The compressive strength of the induration decreases when the water content increases. When water is added, the smooth cell wall shows a convex cell body and lines, resulting in the deterioration of the mechanical strength.

## Figures and Tables

**Table 1:** Physical properties of gangues with different particle sizes.

Particle Size (mm)	15–20	10–15	5–10	0–5
Porosity (%)	48.00	42.29	37.50	35.33
Water absorption (%)	2.11	2.33	2.45	2.83
Water content (%)	1.05	0.85	0.85	0.54
Apparent density (kg/m^3^)	2532	2388	2276	2250
Bulk density (kg/m^3^)	1087	1150	1285	1270

**Table 2:** Absorption bonds of FTIR spectra for PU.

Assignment	Wavenumber (cm^−1^)
Stretching vibration of C-O in aliphatic ether	1071
Stretching vibration of C-O in ester group	1219
δ (N-H) + ν(C-N), amide III band	1306
δ (N-H) + ν(C-N), amide II band	1512
Benzene ring skeleton vibration	1595
Stretching vibration of amido-carbonyl C=O	1712
Stretching vibration of methyl and methylene	2923
N–H stretching vibrations of hydrogen bonding	3300
Stretching vibration of free hydroxy(-OH)	3691

## Data Availability

The data that support the findings of this work are available from the first author on reasonable request.
